# Shapes of Nonsymmetric Capillary Bridges

**DOI:** 10.1021/acs.jpcb.1c07448

**Published:** 2021-10-28

**Authors:** L. R. Pratt, D. T. Gomez, A. Muralidharan, N. Pesika

**Affiliations:** † Department of Chemical and Biomolecular Engineering, Tulane University, New Orleans, Louisiana 70118, United States; ‡ Department of Chemistry, University of Wisconsin—Madison, Madison, Wisconson 53706, United States

## Abstract

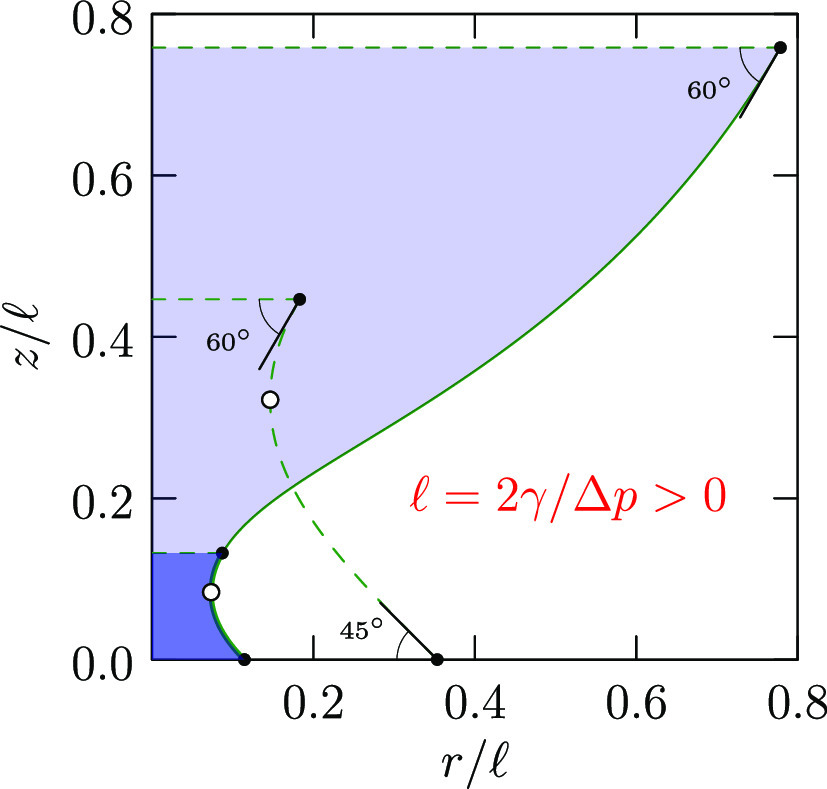

Here we study the shapes of droplets captured between chemically distinct parallel plates. This work is a preliminary step toward characterizing the influence of second-phase bridging between biomolecular surfaces on their solution contacts, i.e., capillary attraction or repulsion. We obtain a simple, variable-separated quadrature formula for the bridge shape. The technical complication of double-ended boundary conditions on the shapes of nonsymmetric bridges is addressed by studying *waists* in the bridge shape, i.e., points where the bridge silhouette has zero derivative. Waists are generally expected with symmetric bridges, but waist points can serve to characterize shape segments in general cases. We study how waist possibilities depend on the physical input to these problems, noting that these formulas change with the *sign* of the inside–outside pressure difference of the bridge. These results permit a variety of different interesting shapes, and the development below is accompanied by several examples.

## Introduction

**Figure 1:**
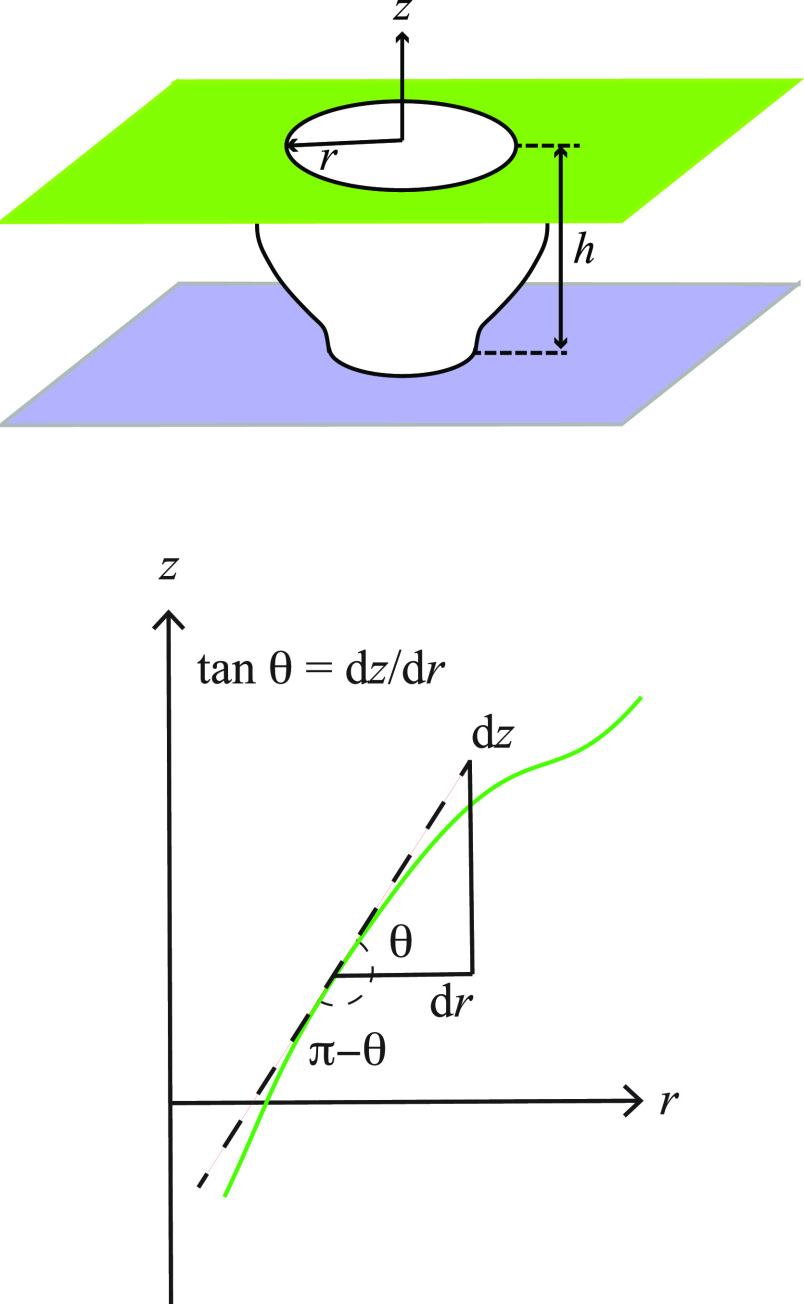
(top) A nonsymmetric capillary bridge studied here and (bottom) angles associated with a general droplet shape.

Here we study the shapes of nonsymmetric capillary bridges between planar contacts (Figure 1), laying a basis for studying the forces that result from the bridging.

The recent measurements of Cremaldi et al.,^1^ provide a specific motivation for this work. A helpful monograph^2^ sketches adhesion due to symmetric capillary bridges, albeit with aspect ratio (width/length ≈10^3^) vastly different than is considered below. Additionally, that sketch^2^ does not specifically consider nonsymmetric cases surveyed by Cremaldi et al.^1^ A specific description applicable to nonsymmetric cases is apparently unavailable^3^ and, thus, is warranted here.

A background aspect of our curiosity in these problems is the possibility of evaporative bridging between ideal hydrophobic surfaces, influencing the solution contacts between biomolecules.^4−10^ Assessment of critical evaporative lengths in standard aqueous circumstances on the basis of explicit thermophysical properties^8^ sets those lengths near 1 μm. Though we do not specifically discuss that topic further here, our analytical development does hinge on identification of the length , with γ the fluid interfacial tension, and Δ*p* the pressure difference between inside and outside of the bridge. The experiments that motivate this study considered spans ≲(6 μL)^1/3^ ≈ 1.8 mm.^1^

A full development of the essential basics of this problem might be dense in statistical-thermodyamics. We strive for concision in the presentation below but follow^2^ a Grand Ensemble formulation of our problem. We then develop the optimization approach analogous to Hamilton’s Principle of classical mechanics.^2,11^ That approach avoids more subtle issues of differential geometry related to interfacial forces and, eventually, should clarify the thermodynamic forces for displacement of the confining plates. Along the way, we support the theoretical development by displaying typical solutions of our formulation.

## Statistical Thermodynamic Formulation

**Figure 2:**
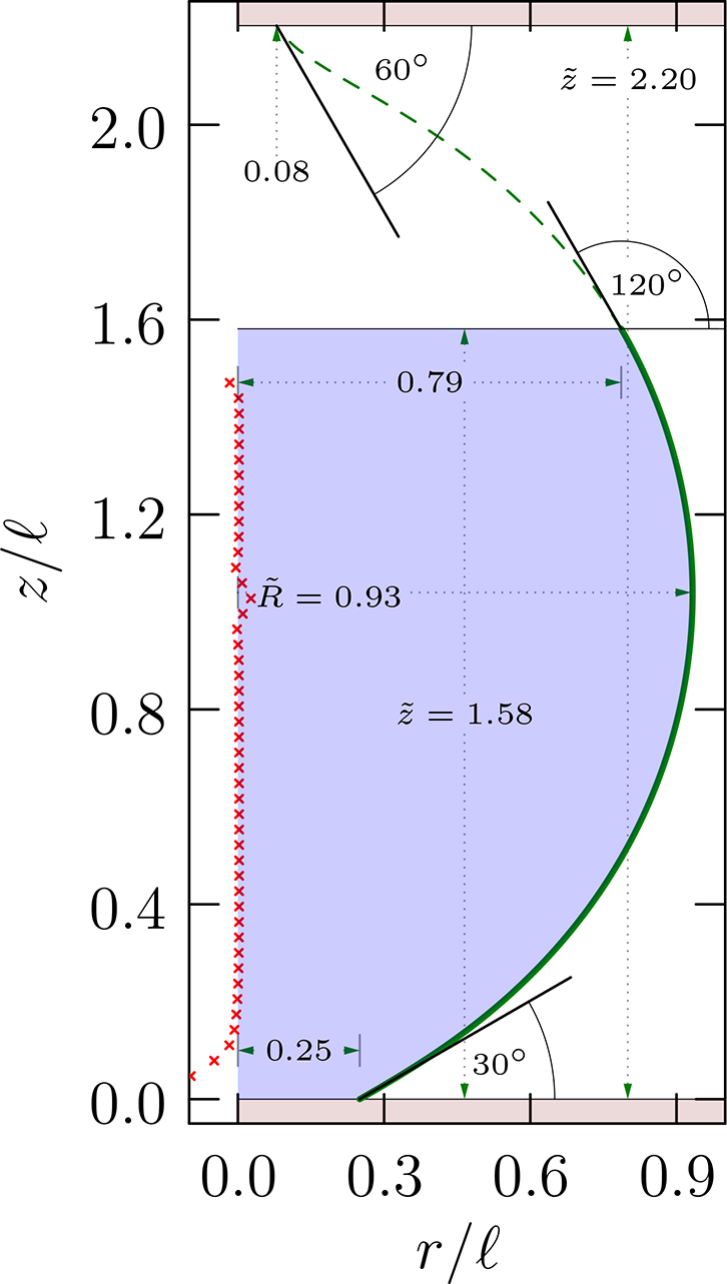
Droplet dimensions using lengths scaled by , with Δ*p* > 0. That the pressure is higher inside, the blue shaded region, than outside the droplet is recognized by noting that *r̈* is negative at the waist. Our separation of variables, eq 18 which is used here, suggests taking *r* (the horizontal axis) as the independent variable. At the bottom contact  in eq 15 with θ = 30°. The waist has radius . The alternative solution of eq 15 is , smaller than the radius of the upper contact, ≈0.08. The contact angle θ_+_ = 60° together with *R̃*, eq 15 gives *r̃*_+_ ≈ 0.79, confirming the connection between branches above and below the waist. The dashed curve thus extends the solid curve. At each height, the red crosses mark the discrepancies of the Euler–Lagrange eq 9 from zero.

Consider two plates, not necessarily the same, oriented perpendicular to the *z*-axis and separated by a distance *h* (Figure 1). A droplet captured between two parallel plates is assumed to be cylindrically symmetric about the *z*-axis. We want to determine the droplet shape (Figure 2) in advance of analyses of the forces involved. We study
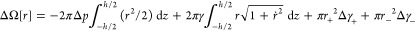
1a functional of the droplet radius *r*(*z*). Here *ṙ* = d*r*(*z*)/d*z* and *r*_±_ = *r*(*z* = ±*h*/2). γ is the tension between the droplet and the external solution. Δγ_+_ is the inside–outside difference of the surface tensions of the fluids against the plate at *z* = +*h*/2 (and similarly for Δγ_–_ with the fluids against the plate at *z* = −*h*/2); this differencing will be clarified below as we note how this leads to Young’s Law. Δ*p* is the traditional Laplace inside–outside pressure difference of the bridge. The usual Grand Ensemble potential for a single-phase uniform fluid solution being −Ω = *pV*, it is natural that ΔΩ[*r*] of eq 1 has Ω for the surrounding fluid solution subtracted away; *i.e*, the pressure–volume term of eq 1 evaluates the pressure inside times the bridge volume, *minus* the pressure outside times the same bridge volume. Formally
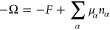
2with *F* the Helmholtz free energy. Therefore, the surface-area feature of eq 1 can be viewed as an addition of *γA* contribution to *F*, with *A* the surface area of contact of the bridge with the external fluid and γ is the tension of the fluid–fluid interface. We have not included a line tension associated with the top/bottom contacts. Nevertheless, since eq 1 is firmly grounded in the basic physical description of our problem, line tension issues should be readily accommodated.

An alternative perspective on ΔΩ (eq 1) is that it is a Lagrangian function for finding a minimum surface area of the bridge satisfying a given value of the bridge volume. Then Δ*p*/γ, which has dimensions of an inverse length, serves as a Lagrange multiplier. We then minimize ΔΩ with respect to variations of *r*(*z*), targeting a specific value of the droplet volume.

The first-order variation of ΔΩ is then
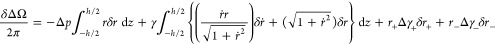
3

The angle that the shape curve *r*(*z*) makes with the plane perpendicular to the *z* axis (Figure 1) is
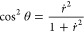
4and at the contacting surfaces
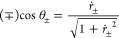
5

Depicted in Figure 1 is the choice of the bottom sign above, where 0 < *ṙ* < ∞. For θ_+_ we change the choice so that the contact angle at the upper plate is the traditional external angle of the droplet.

The usual integration-by-parts for eq 3 gives
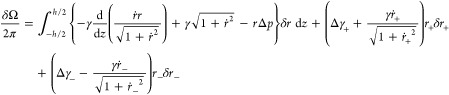
6

With the signs indicated in eq 5
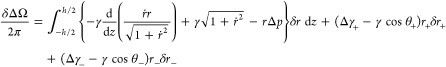
7with the exterior angles contacting the upper and lower plates.

The contact terms in eq 7 vanish if the contact angles obey the force balance
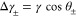
8of the traditional Young’s Law. This reinforces the sign choice for eq 5. Equation 8 will provide boundary information for *r*(*z*).

From eq 7, we require that the kernel
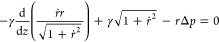
9vanish identically in *z*. As with Young’s Law, this balances the forces for varying the droplet radius. For the example of a spherical droplet of radius *R*, this force balance implies the traditional Laplace pressure formula, Δ*p* = 2γ/*R*.

The traditional Hamilton’s principle^11^ analysis of this formulation then yields the usual energy conservation theorem^2,11^
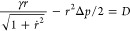
10with *D* a constant of integration. *D* + *r*^2^Δ*p*/2 is non-negative according to eq 10. Recognizing that sign, then
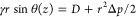
11with 0 ≤ θ(*z*) ≤ π. The constant *D* can be eliminated in terms of boundary information, e.g.,
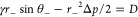
12

This helpfully correlates *r*(*z*) at other places too. For example, we will consider (Figure 2) intermediate positions where *ṙ*(*z*) = 0 and sin θ(*z*) = 1. We call such a position a “waist”. A waist is expected for symmetric cases that we build from here. Denoting the radius of a waist by *R*, then
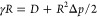
13from eq 10. This eliminates the integration constant *D* in favor of *R*, which may be more meaningful.
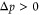


Considering Δ*p* > 0 we can make these relations more transparent by nondimensionalizing them with the length . Then  and , so
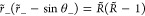
14

**Figure 3:**
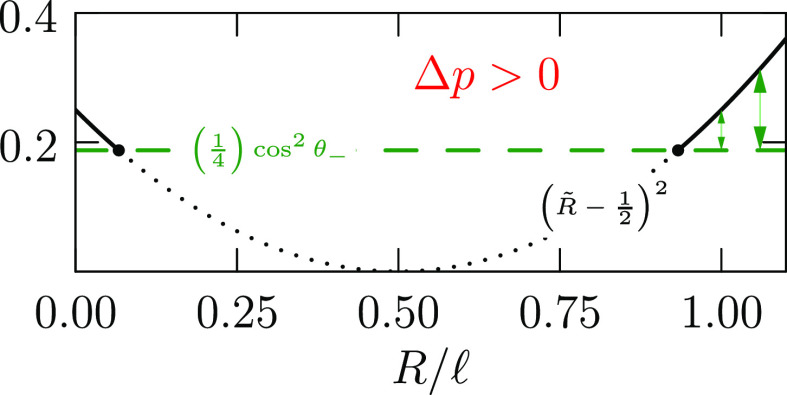
For contact angle θ_–_, eq 15 requires that . Thus, the solid black curves cover possible values of *R̃* for this θ_–_, and displacements upward from the green horizontal line, i.e., the arrows, show values of (*r̃*_–_ – sin θ_–_/2)^2^. The θ_–_ adopted for this drawing is π/6 as for the bottom branch shown in Figure 2, and the right-most dot locates the value of the waist radius there (Figure 2). Thus, the waist in that example is the slimmest waist in that range. Such considerations apply to both top and bottom contacts with their distinct contact angles. A contact angle near π/2 will correspond to a lower level for the horizontal line and thus be less restrictive of the possible values of a common waist radius *R̃*.

Though this scaling with the length  is algebraically convenient, Δ*p* can take different signs in different settings; indeed, calculating from eq 9, at a waist Δ*p*/γ = 1/*R* – *r̈* in the present setup, with *r̈* the curvature at that waist. Completing the square from eq 14 gives
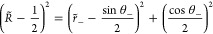
15Equation 15 provides helpful perspective (Figure 3) for exploring different bridge sizes. Given θ_–_, this requires that (*R̃* – 1/2)^2^ ≥ (cos θ_–_/2)^2^, as is evident there.

Interesting further consequences follow from considerations of the cases that the droplet is nearly tangent to the contact surfaces: θ_±_ = 0 or π. Consider first θ_–_ → 0. The droplet approaches detachment from the lower surface. We expect *r*_–_ → 0 then. Figure 3 shows that this can be achieved with *R̃* = 0 or 1. The *R̃* = 1 case produces a hemispherical lower portion on the bridge, with the hemisphere just touching the lower surface and  from eq 15.

When θ_+_ → π for example, the droplet preferentially wets the upper surface. We expect *r*_+_ to be relatively large then, and this force contribution describes interplate attraction, though not necessarily with a waist.

### More Generally but Δ*p* ≠ 0

Restoring in eqs 14 and 15 the dependence on  for Δ*p* ≠ 0, though possibly negative, then gives
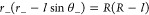
16a
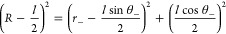
16b is a *signed* length here. With these notations,
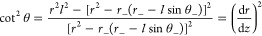
17and
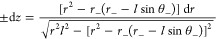
18separates these variables for integration.

**Figure 4:**
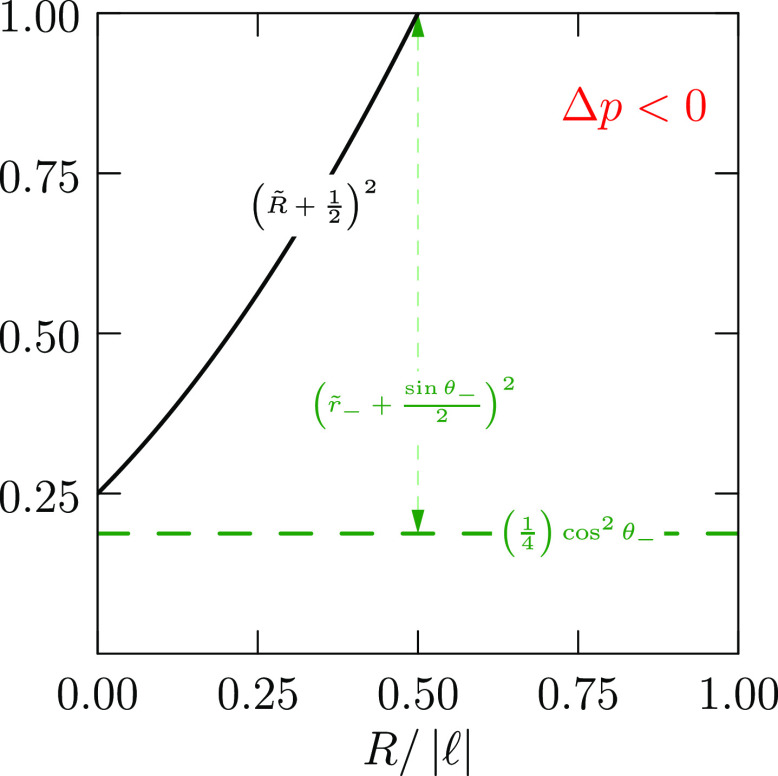
Analogue of Figure 3 but for the case Δ*p* < 0. See eq 19.

We can still follow scaled lengths  and . Then the analogue of eq 15 is
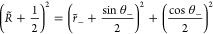
19when Δ*p* < 0; see Figure 4. The analogue of eq 18 with this length scaling for Δ*p* < 0 is
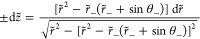
20

To achieve Δ*p*/γ = 1/*R* – *r̈* < 0 for a bridge with wiast radius *R*, clearly the curvature *r̈* at that waist should be substantially positive to ensure that the negative second contribution dominates. In addition, the radius at the waist should be fairly large, thereby reducing the contribution of the positive first term. These points combined suggest that to achieve adhesion the contact areas should be larger than the waist area, which itself should be substantial.

### Waist *R*

Reaffirming the identification of *R* as the radius of a waist and specifically recalling that  is a *signed* length:
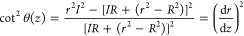
21Factoring out the cot^2^ θ(*r̃*^2^ = *R̃*^2^) = 0 feature gives
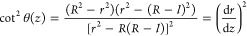
22eq 22 also shows that cot^2^ θ(*z*) = 0 at the point .

Equation 22 then achieves the separation of variables
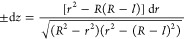
23for integration in this case.

## Examples

In the example Figure 2 (Δ*p* > 0), *R̃* ≈ 0.933 and (*R̃* – 1)^2^ ≈ 0.067^2^, smaller than the radius of the upper cross-section, 0.08^2^, in that extended example. The slimmer *second waist* is not realized.

**Figure 5:**
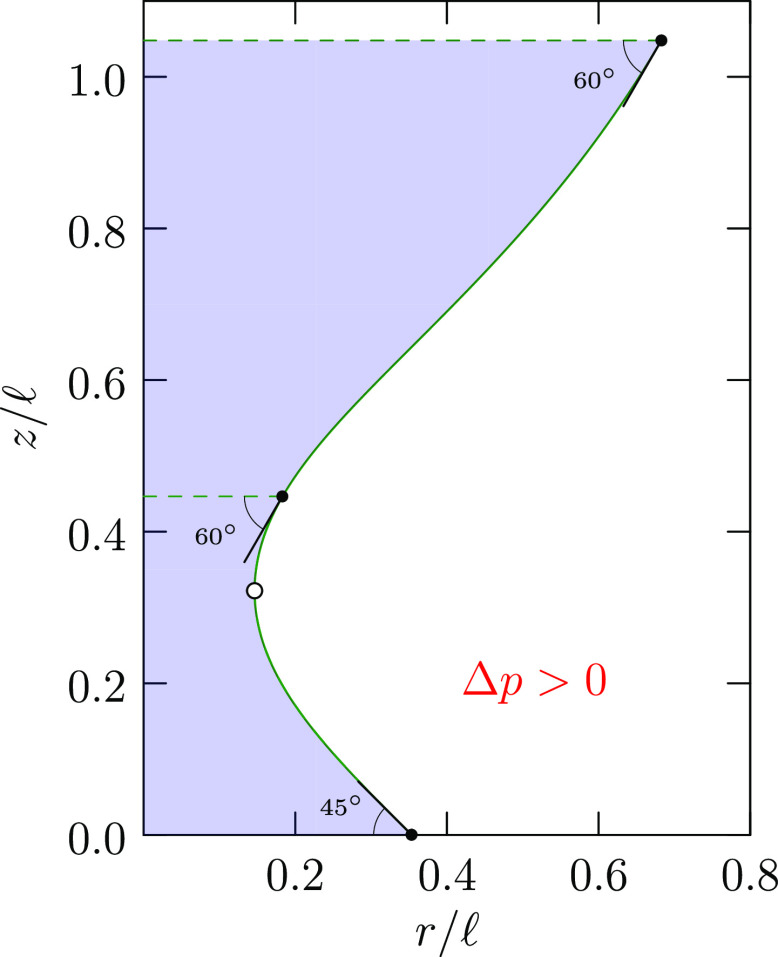
Capillary bridge shape for the biggest slim-waisted possibility of Figure 6. Here the pressure inside is greater than the pressure outside, so eq 18 is used. The open circle marks the waist. Δ*p*/γ = 1/*R* – *r̈* at a waist of radius *R*, so achieving Δ*p* > 0 with positive curvature *r̈*, as above, limits the waist radius *R*. This solution exhibits the upper contact angle twice.

**Figure 6:**
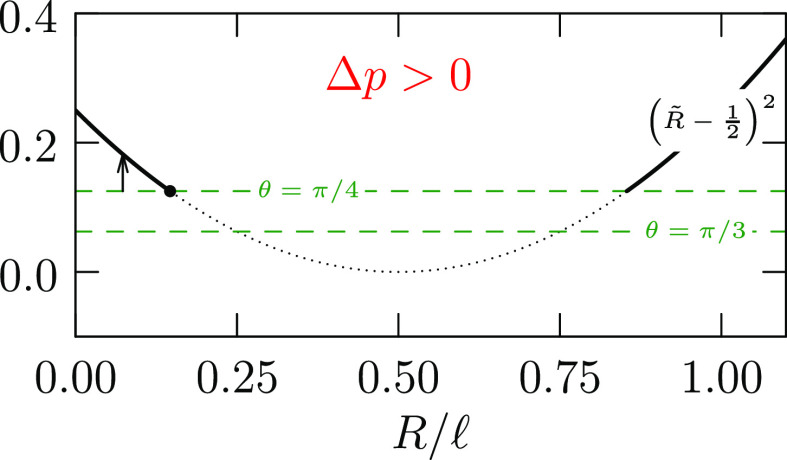
Considerations for choice of waist radii *R̃* for the slim-waisted bridge of Figure 5.

Figure 5 shows a bridge shape for the slender waist identified for the contact angles specified in Figure 6 for Δ*p* > 0.

**Figure 7:**
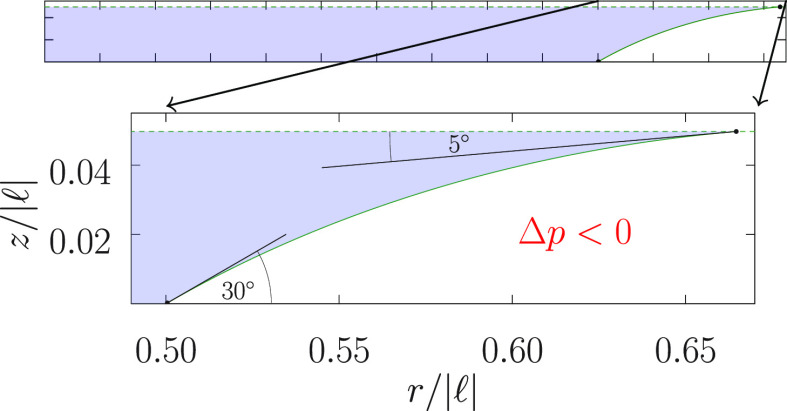
From eq 23, with the indicated contact angles and with Δ*p* < 0, so that pressure inside the bridge is less than the pressure outside. *R̃* ≈ 0.366, from eq 19 and Figure 4. Since the smallest contact radius—at the bottom plate—is 0.5, the waist at *R̃* ≈ 0.366 is not realized in this physical range.

In the example Figure 7, Δ*p* < 0 and . Thus, , and the (−) of eq 23 is required to achieve a positive slope at the bottom plate. The aspect ratio of the bridge is vastly changed, as was true also in the discussion of capillary adhesion of ref (2); capillary adhesion would be expected for this shape.

## Discussion

In view of the variety of interesting shape possibilities, we reserve explicit study of the consequent interplate forces, and of the stability/metastability of these bridges, for a specific experimental context. Nevertheless, we outline here how such a practical study might be implemented.

The setup above permits straightforward calculation of the thermodynamic potential Ω and
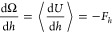
24*U* being the internal energy, positive values of *F*_*h*_ indicate that *U* decreases with increasing *h*, the temperature being constant in these considerations. Thus, positive values of *F*_*h*_ indicate repulsion, and negative values describe attraction.

Our motivating example is Cremaldi et al.;^1^ in those cases a waist with radius *R̃* is clear, and we anticipate that Δ*p* > 0. To connect to specific experimental cases, we note that *a priori* experimental data are γ, the contact angles θ_–_ and θ_+_, the experimental volume of the captured droplet *v*, and interplate separation *h*. Equation 15 and Figure 3 show permitted ranges for *R̃*. With these parameters set, integration (eq 23) determines Δ*z̃* = *z̃*_+_ – *z̃*_–_. Then
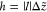
25so that
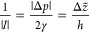
26matching the experimental *h*. [What is more, the *sign* of Δ*p* is known through the calculational procedure.] We then further evaluate the volume of droplet
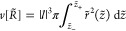
27as it depends on *R̃*, and seek a match with the experimental droplet volume *v*. If *R̃* were provided *a priori*, eqs 26 and 27 would overdetermine . But *R̃* is not provided *a priori*, so those two equations determine the two remaining parameters  and *R̃*. Since the dependence on  is clear, we can proceed further to
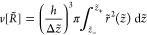
28leaving finally
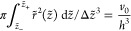
29to be solved for *R̃*.

## Conclusions

We provide general, simple, variable-separated quadrature formulas (eq 23) for the shapes of capillary bridges, not necessarily symmetric. The technical complications of double-ended boundary conditions on the shapes of nonsymmetric bridges are addressed by studying *waists* in the bridge shapes, noting that these relations change distinctively with the change-of-sign of the inside–outside pressure difference of the bridge (eq 16b). These results permit a variety of different interesting cases, and we discuss how these analyses should be implemented to study forces resulting from capillary bridging between neighboring surfaces in solutions.

## References

[ref1] Cremaldi J. C.; Khosla T.; Jin K.; Cutting D.; Wollman K.; Pesika N. Interaction of Oil Drops with Surfaces of Different Interfacial Energy and Topography. Langmuir 2015, 31, 3385–3390. 10.1021/acs.langmuir.5b00051. 25723337

[ref2] deGennes P.-G.; Brochard-Wyart F.; Quéré D. Capillarity and Wetting Phenomena: Drops, Bubbles, Pearls, Waves; Springer Science & Business Media, 2013.

[ref3] Lv C.; Shi S. Wetting states of two-dimensional drops under gravity. Phys. Rev. E: Stat. Phys., Plasmas, Fluids, Relat. Interdiscip. Top. 2018, 98, 042802–10. 10.1103/PhysRevE.98.042802.

[ref4] Wallqvist A.; Gallicchio E.; Levy R. M. A Model for Studying Drying at Hydrophobic Interfaces: Structural and Thermodynamic Properties. J. Phys. Chem. B 2001, 105, 6745–6753. 10.1021/jp010945i.

[ref5] Huang X.; Margulis C. J.; Berne B. J. Dewetting-induced collapse of hydrophobic particles. Proc. Natl. Acad. Sci. U. S. A. 2003, 100, 11953–11958. 10.1073/pnas.1934837100. 14507993 PMC218694

[ref6] Huang X.; Margulis C. J.; Berne B. J. Correction for Huang et al., Dewetting-induced collapse of hydrophobic particles. Proc. Natl. Acad. Sci. U.S.A. 2006, 103, 19605–19605. 10.1073/pnas.0610370103. PMC218694 14507993

[ref7] Choudhury N.; Pettitt B. M. On the Mechanism of Hydrophobic Association of Nanoscopic Solutes. J. Am. Chem. Soc. 2005, 127, 3556–3567. 10.1021/ja0441817. 15755177

[ref8] Cerdeiriña C. A.; Debenedetti P. G.; Rossky P. J.; Giovambattista N. Evaporation Length Scales of Confined Water and Some Common Organic Liquids. J. Phys. Chem. Lett. 2011, 2, 1000–1003. 10.1021/jz200319g.

[ref9] Dzubiella J.; Swanson J.; McCammon J. Coupling nonpolar and polar solvation free energies in implicit solvent models. J. Chem. Phys. 2006, 124, 084905 10.1063/1.2171192. 16512740

[ref10] Bharti B.; Rutkowski D.; Han K.; Kumar A. U.; Hall C. K.; Velev O. D. Capillary bridging as a tool for assembling discrete clusters of patchy particles. J. Am. Chem. Soc. 2016, 138, 14948–14953. 10.1021/jacs.6b08017. 27775335

[ref11] Goldstein S. Classical Mechanics; Addison-Wesley: Reading, MA, 1950; Chapter 2.

